# Supplementary material for: Deletion of wfs1 Impairs Oligodendrocyte Precursor Cells Dorsal Distribution and Myelination Through the wfs1-hmgcs1 Axis in Zebrafish
Source: Biology (Basel). 2026 Aug 16;15(16):1402. doi: 10.3390/biology15161402 (PMC13510151; doi:10.3390/biology15161402)
Supplement: Supplementary file 1 [file biology-15-01402-s001.zip › File S1-Transcriptome sequencing data and differentially expressed genes (DEGs) in wfs1-- zebrafish at 5 dpf/2.KEGG/wfs1KOvsWT/DOWN/src/dre00010.html]

dre00010


- dre:100005587

- Down regulated genes

100005587\_1(-1.54424530950344)
- dre:100536795

- Down regulated genes

100536795(-1.64304185838866)
- dre:100006238

- Down regulated genes

100006238\_1(-1.31531604695891)

- dre:445326

- Down regulated genes

445326(-1.62876055255802)

- dre:393427

- Down regulated genes

393427(-1.25663853074503)

- dre:560753

- Down regulated genes

560753(-1.42485234049394)

- dre:568001

- Down regulated genes

568001\_2(-2.92834331323733)
- dre:561416

- Down regulated genes

561416(-4.80431336765849)

- dre:406339

- Down regulated genes

406339\_1(-1.38680349052238)

- dre:436646

- Down regulated genes

436646(-2.66783103485591)

- dre:406339

- Down regulated genes

406339\_1(-1.38680349052238)

Close
